# Supplementary material for: Effect of Multiply Twinned Ag(0) Nanoparticles on Photocatalytic Properties of TiO2 Nanosheets and TiO2 Nanostructured Thin Films
Source: Nanomaterials (Basel). 2022 Feb 23;12(5):750. doi: 10.3390/nano12050750 (PMC8911912; doi:10.3390/nano12050750)
Supplement: Supplementary file 1 [file nanomaterials-12-00750-s001.zip › nanomaterials-1567189-supplementary.pdf]

Supplementary Information

Effect of Multiply Twinned $Ag^{(0)}$ Nanoparticles on Photocatalytic Properties of TiO_2 Nanosheets and TiO_2 Nanostructured Thin Films

Snejana Bakardjieva [1,2,*], Jakub Mares [1,2], Eva Koci [1], Jakub Tolasz [1], Radek Fajgar [3], Vasyl Ryukhtin [4], Mariana Klementova [5], Štefan Michna [2], Hana Bibova [6], Randi Holmestad [7], Rositsa Titorenkova [8] and Maria Caplovicova [9]

1 Institute of Inorganic Chemistry of the Czech Academy of Sciences, 250 68 Husinec-Rez, Czech Republic; mares@iic.cas.cz (J.M.); evulekotule@iic.cas.cz (E.K.); jtolasz@iic.cas.cz (J.T.)
2 Faculty of Mechanical Engineering, JE Purkyně University, Pasteurova 1, 400 96 Ústí nad Labem, Czech Republic; stefan.michna@ujep.cz
3 Institute of Chemical Process and Fundamentals of the Czech Academy of Sciences, Rozvojova 2/135, 165 02 Prague, Czech Republic; fajgar@icpf.cas.cz
4 Nuclear Physics Institute of Czech Academy of Sciences, 250 68 Husinec-Rez, Czech Republic; ryukhtin@ujf.cas.cz
5 Institute of Physics of the Czech Academy of Sciences, Na Slovance 1999/2, 182 21 Prague, Czech Republic; klemari@fzu.cz
6 J. Heyrovsky Institute of Physical Chemistry of the Czech Academy of Sciences, Dolejskova 2155/3, 182 23 Prague, Czech Republic; hana.bibova@jh-inst.cas.cz
7 Department of Physics, Norwegian University of Science and Technology (NTNU), NO 7491 Trondheim, Norway; randi.holmestad@ntnu.no
8 Institute of mineralogy and crystallography, Bulgarian Academy of Sciences, 107, Acad. G. Bonchev Str., 1113 Sofia, Bulgaria; rositsatitorenkova@imc.bas.bg
9 STU Centre for Nanodiagnostics, University Science Park Bratislava Centre, Slovak University of Technology, Vazovova 5, 811 07 Bratislava, Slovakia; maria.caplovicova@stuba.sk
* Correspondence: snejana@iic.cas.cz

Citation: Bakardjieva, S.; Mares, J.; Koci, E.; Tolasz, J.; Fajgar, R.; Ryukhtin, V.; Klementova, M.; Michna, Š.; Bibova, H.; Holmestad, R.; et al. Effect of Multiply Twinned $Ag^{(0)}$ Nanoparticles on Photocatalytic Properties of TiO_2 Nanosheets and TiO_2 Nanostructured Thin Films. *Nanomaterials* **2022**, *12*, 750. https://doi.org/10.3390/nano12050750

Academic Editors: Kyungsu Na and Marianna Kemell

Received: 10 January 2022
Accepted: 17 February 2022
Published: 23 February 2022

Publisher's Note: MDPI stays neutral with regard to jurisdictional claims in published maps and institutional affiliations.

Copyright: © 2022 by the authors. Licensee MDPI, Basel, Switzerland. This article is an open access article distributed under the terms and conditions of the Creative Commons Attribution (CC BY) license (https://creativecommons.org/licenses/by/4.0/).

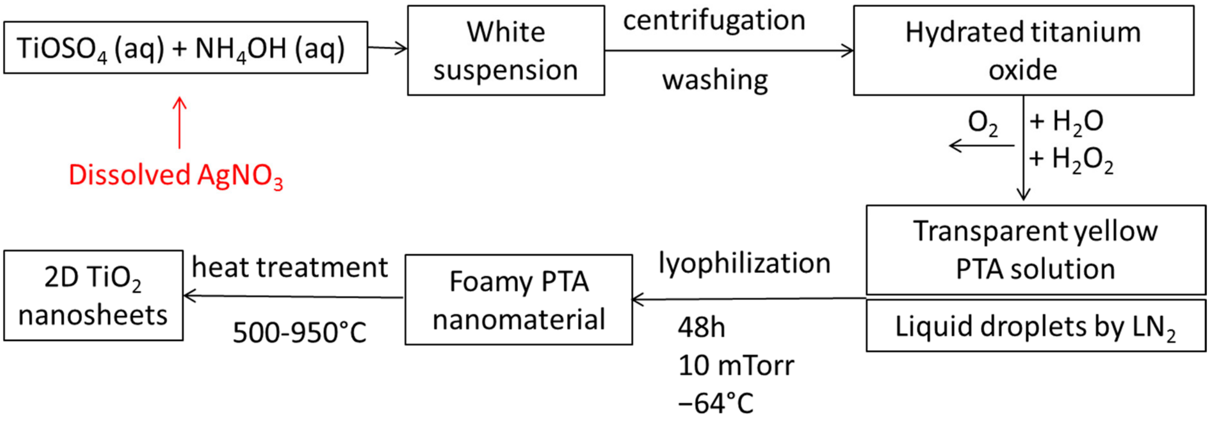

Figure S1. Synthetic route for the preparation of freeze-dried precursor for Ag-modified TiO_2 nanosheets.

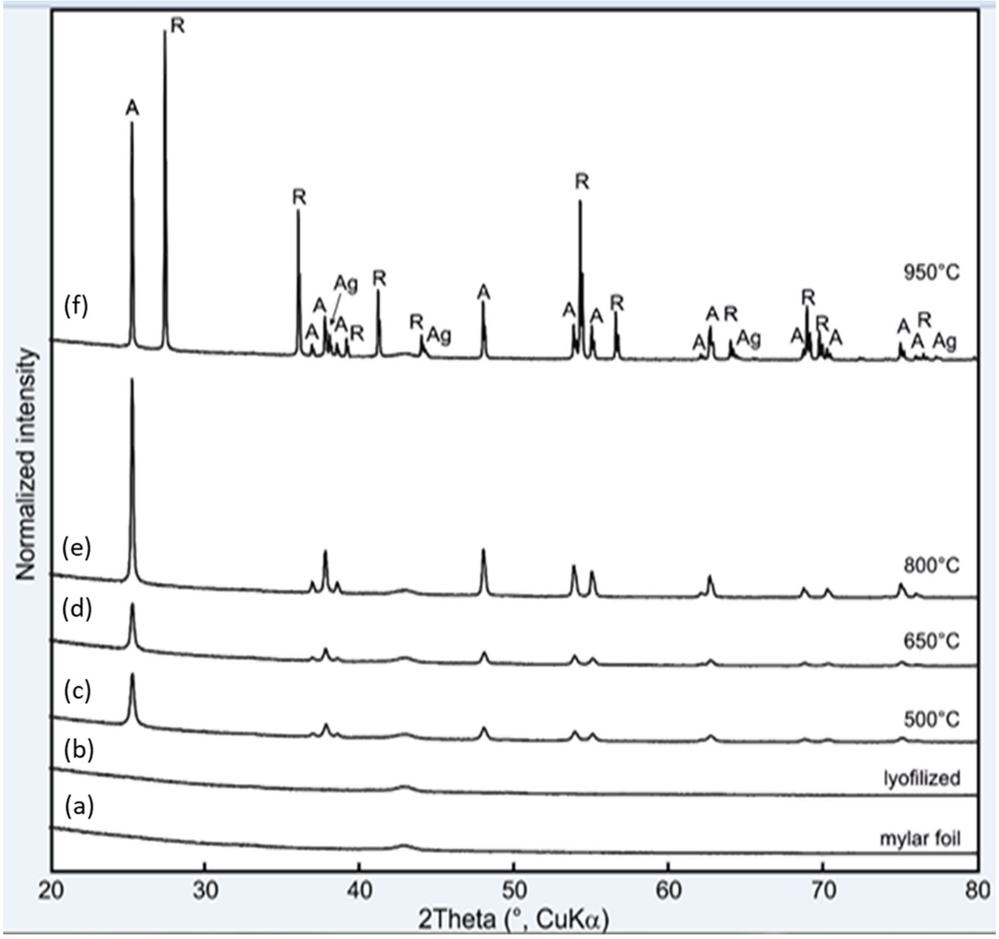

Figure S2. Powder XRD diffraction patterns of Ag modified 2D TiO2 nanosheets: (a) Mylar foil, (b) lyophilized precursor Ag_TiO_LYO, (c) Ag_TiO_LYO/500 obtained at 500 °C, Ag_TiO_LYO/650 obtained at 650 °C, Ag_TiO_LYO/800 obtained at 800 °C, and Ag_TiO_LYO/950 obtained at 950 °C.

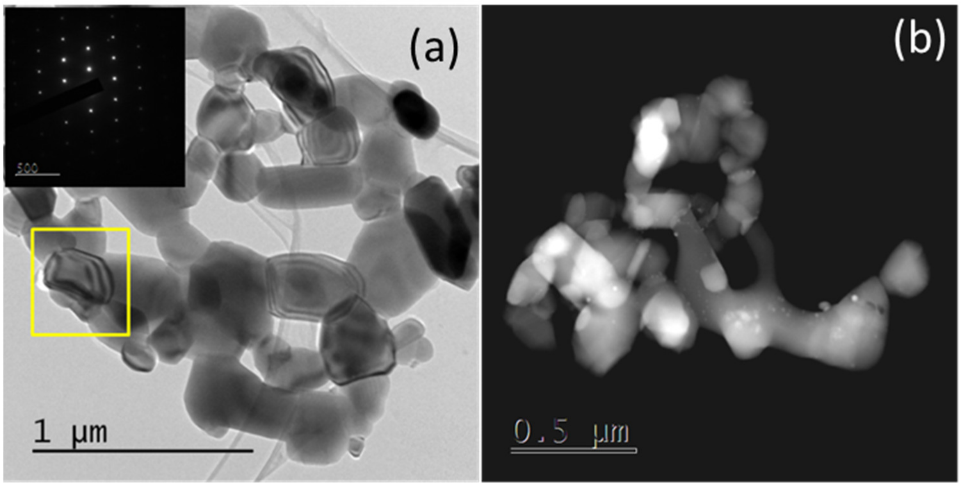

Figure S3. S/TEM images of (a) of Ag_TiO_LYO/950 with SAED inset confirming single nanocrystals of rutile, (b) HAADF image revealed $Ag^{(0)}$ NPs on the surface of TiO_2.

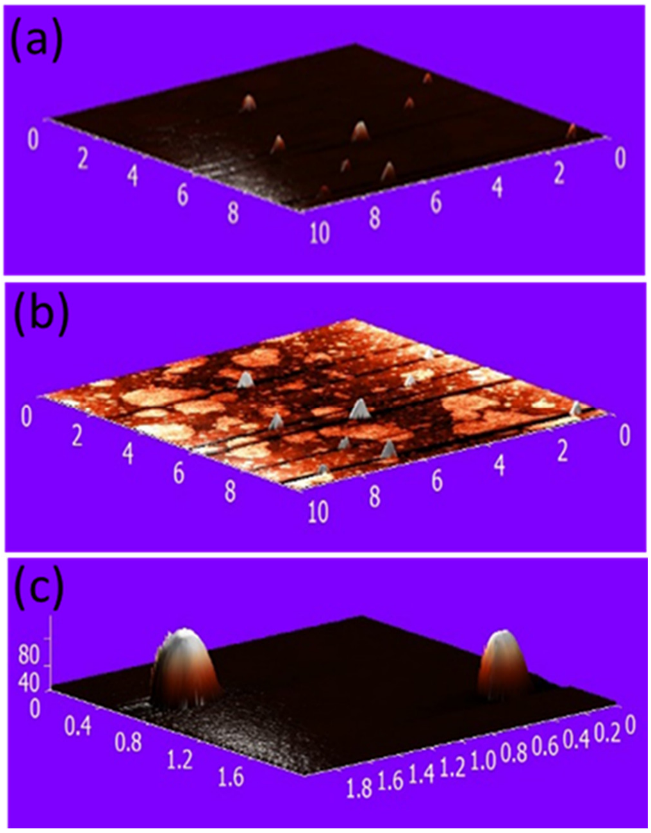

Figure S4. AFM 3D-images of the Ag_TiO_LYO/800 sample deposited on a glass substrate. (a) an image of the sample area of 10 µm × 10 µm with an offset of Z-axis zero setting that allows distinguishing only large nanoparticles, (b) the same image with an offset of Z-axis zero setting that allows distinguishing the nanostructure details with low height, (c) an image of the sample area with a size of 2 µm × 2 µm including only two nanoparticles.

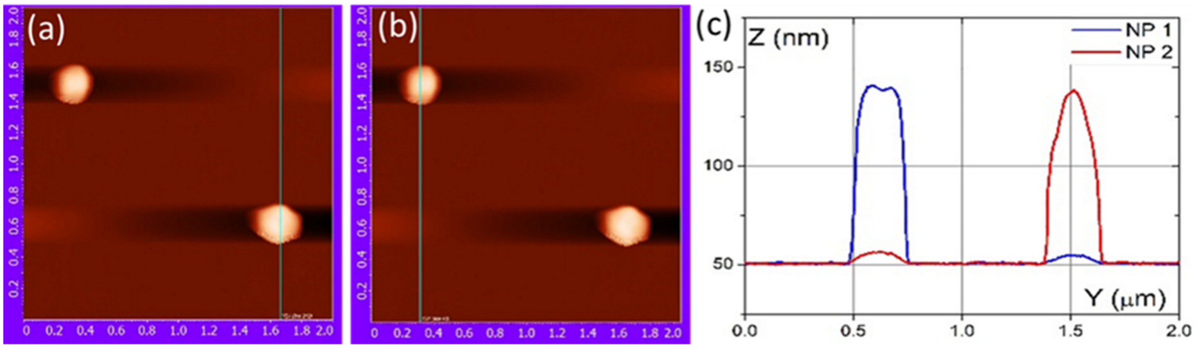

Figure S5. Profiling analysis of the 2D AFM image of the Ag_TiO_LYO/800 deposited on a glass substrate with a size of 2 μm × 2 μm including two NPs. (a) the image with Y-profiling along the right NP1, (b) the image with Y-profiling along the left NP2, (c) the plot of the profiles reflecting the size and height of the NP1 and NP2.

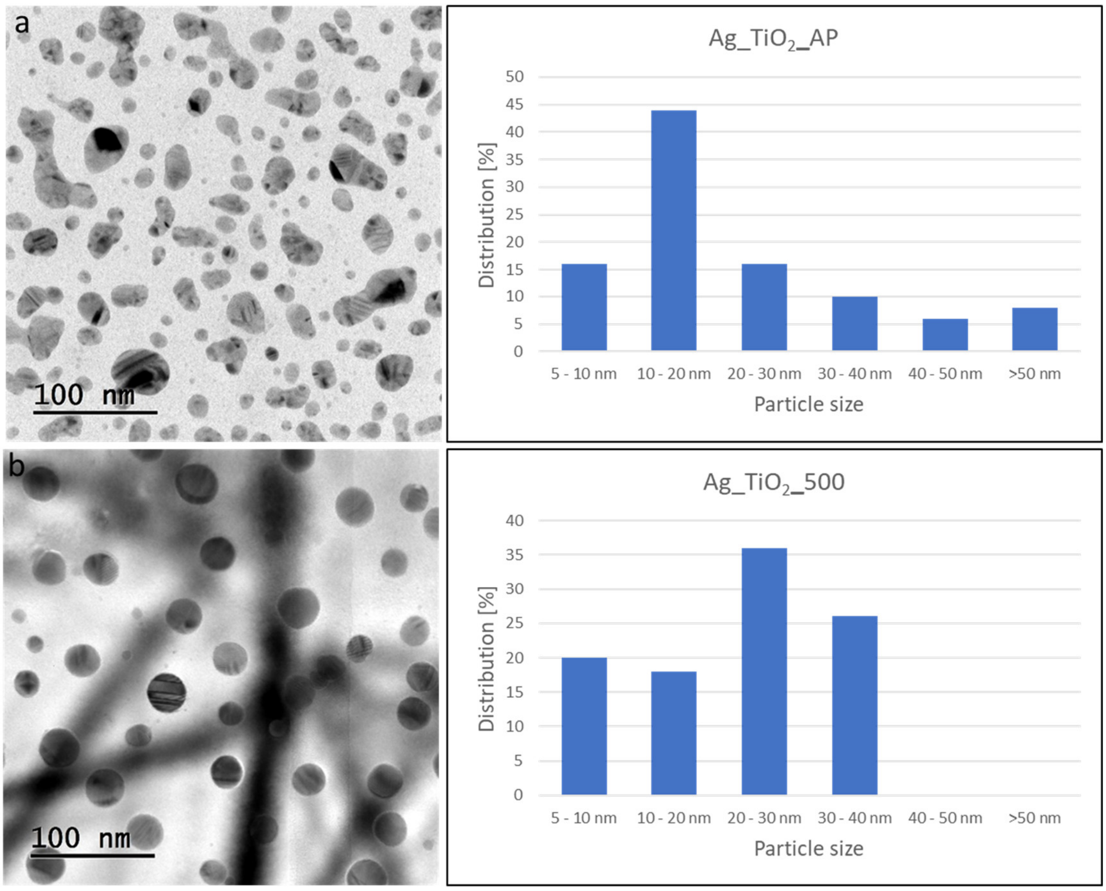

Figure S6. S/TEM image and distribution of nanoparticle size of NSTFs. (a) S/TEM image of Ag_TiO_2_AP with the corresponding distribution of nanoparticle size and (b) S/TEM image Ag_TiO_2_500 NSTF with the corresponding distribution of nanoparticle size obtained by ImageJ software.
